# Supplementary material for: APOE genotype-specific methylation patterns are linked to Alzheimer disease pathology and estrogen response
Source: Transl Psychiatry. 2024 Feb 29;14:129. doi: 10.1038/s41398-024-02834-x (PMC10904829; doi:10.1038/s41398-024-02834-x)
Supplement: Supplementary file 1 — Supplemental Materials [file 41398_2024_2834_MOESM1_ESM.docx]

**SUPPLEMENTARY INFORMATION**

**Supplementary Table 1.** Methylation Array Sample Information

| **Dataset** | **APOE**  **Genotype** | **AD Cases** | | |  | **Controls** | | |
| --- | --- | --- | --- | --- | --- | --- | --- | --- |
|  |  | **N** | **% Female** | **Age** |  | **N** | **% Female** | **Age** |
| ROSMAP | ɛ4 non-carriers | 265 | 69.4 | 90.2 ± 5.7 |  | 246 | 58.9 | 86.4 ± 7.3 |
|  | ɛ4 carriers | 152 | 62.5 | 87.8 ± 5.8 |  | 34 | 55.9 | 84.6 ± 6.7 |
|  | TOTAL | 417 | 66.8 | 89.3 ± 5.9 |  | 280 | 58.6 | 86.1 ± 7.2 |
| ADNI | ɛ4 non-carriers | 29 | 34.5 | 78.3 ± 7.1 |  | 158 | 50.0 | 74.9 ± 5.9 |
|  | ɛ4 carriers | 62 | 35.5 | 72.9 ± 7.0 |  | 52 | 50.0 | 74.1± 5.4 |
|  | TOTAL | 91 | 35.2 | 74.6 ± 7.4 |  | 210 | 50.0 | 74.7 ± 5.8 |
| FHS | ɛ4 non-carriers | NA | NA | NA |  | 1115 | 45.9 | 45.9 ± 8.2 |
|  | ɛ4 carriers | NA | NA | NA |  | 276 | 50.4 | 45.7 ± 8.5 |
|  | TOTAL | NA | NA | NA |  | 1391 | 52.3 | 45.8 ± 8.2 |

ROSMAP: Religious Orders Study and Rush Memory and Aging Project; ADNI: Alzheimer’s Disease Neuroimaging Initiative; FHS: Framingham Heart Study. Methylation array data were generated from autopsied brains in the ROSMAP and blood in the ADNI and FHS. Age = age at exam for individuals in the FHS and ADNI datasets and age of death for individuals in the ROSMAP dataset.

**Supplementary Table 2.** Association of AD differentially methylated CpG Sites (P<10^-5^) from *APOE* ɛ4 carriers and non-carriers in brain with tangle and plaque pathology

| **CpG Name** | **Chr** | **Position** | **Gene** | **Braak Stage** | |  | **CERAD Score** | |
| --- | --- | --- | --- | --- | --- | --- | --- | --- |
|  |  |  |  | **T** | **P** |  | **T** | **P** |
| cg19533050 | 2 | 163175044 | IFIH1 | -1.26 | 0.21 |  | 0.22 | 0.82 |
| cg23808213 | 2 | 166948291 | SCN1A | -2.74 | 6.3x10^-3^ |  | 3.73 | 2.1x10^-4^ |
| cg05731218 | 2 | 216769199 | intergenic | -6.99 | 6.6x10^-12^ |  | 6.10 | 1.8x10^-9^ |
| cg04436449 | 3 | 185214835 | TMEM41A | -2.66 | 7.9x10^-3^ |  | 3.42 | 6.6x10^-4^ |
| cg12307200 | 3 | 188664632 | intergenic | -7.05 | 4.2x10^-12^ |  | 7.36 | 5.1x10^-13^ |
| cg16234490 | 4 | 77138082 | FAM47E | -2.78 | 5.6x10^-3^ |  | 3.54 | 4.3x10^-4^ |
| cg24899806 | 7 | 119914282 | KCND2 | -2.15 | 0.03 |  | 3.28 | 1.1x10^-3^ |
| cg23831517 | 8 | 34182528 | intergenic | 2.69 | 7.4x10^-3^ |  | -3.51 | 4.7x10^-4^ |
| cg14096074 | 9 | 34255149 | KIF24 | -2.14 | 0.03 |  | 3.34 | 8.7x10^-4^ |
| cg03727169 | 10 | 31418969 | intergenic | -2.81 | 5.1x10^-3^ |  | 2.54 | 0.01 |
| cg01982597 | 10 | 50733420 | ERCC6 | -3.43 | 6.3x10^-4^ |  | 3.26 | 1.1x10^-3^ |
| cg20326704 | 10 | 70321770 | TET1 | -1.86 | 0.06 |  | 0.86 | 0.39 |
| cg04126866 | 10 | 85932763 | C10orf99 | -3.89 | 1.1x10^-4^ |  | 4.43 | 1.1x10^-5^ |
| cg14882481 | 11 | 107437051 | ALKBH8 | -2.16 | 0.03 |  | 4.02 | 6.4x10^-5^ |
| cg10907744 | 12 | 131589455 | GPR133 | 4.57 | 5.8x10^-6^ |  | -4.62 | 4.6x10^-6^ |
| cg18708502 | 13 | 21588555 | LATS2 | 2.95 | 3.3x10^-3^ |  | -3.38 | 7.7x10^-4^ |
| cg16746221 | 14 | 20666088 | OR11G2 | -2.59 | 0.01 |  | 3.03 | 2.5x10^-3^ |
| cg24231804 | 15 | 67316861 | intergenic | -3.07 | 2.2x10^-3^ |  | 4.27 | 2.2x10^-5^ |
| cg14829066 | 15 | 88559141 | NTRK3 | 3.33 | 9.2x10^-4^ |  | -4.41 | 1.2x10^-5^ |
| cg19987111 | 15 | 101747167 | CHSY1 | -5.41 | 8.5x10^-8^ |  | 4.39 | 1.3x10^-5^ |
| cg02432274 | 16 | 88378468 | intergenic | 2.53 | 0.01 |  | -1.45 | 0.15 |
| cg05952786 | 17 | 48559485 | RSAD1 | -2.42 | 0.02 |  | 3.96 | 8.4x10^-5^ |
| cg15503752 | 17 | 74639731 | ST6GALNAC1 | 3.94 | 9.1x10^-5^ |  | -3.56 | 4.0x10^-4^ |
| cg05421550 | 19 | 4446485 | UBXN6 | -1.53 | 0.13 |  | 3.89 | 1.1x10^-4^ |
| cg19612770 | 19 | 4475216 | HDGF2 | -2.74 | 6.4x10^-3^ |  | 3.87 | 1.2x10^-4^ |

T: T-value, P: P-value

**Supplementary Table 3.** AD differentially methylated CpG Sites (P<10^-5^) from *APOE* ε4 carriers and non-carriers in blood

| **CpG Name** | **Chr** | **Position** | **Gene** | **Total Sample** | |  | ***APOE* ε4 carriers** | |  | ***APOE* ε4 non-carriers** | |
| --- | --- | --- | --- | --- | --- | --- | --- | --- | --- | --- | --- |
|  |  |  |  | **T** | **P** |  | **T** | **P** |  | **T** | **P** |
| cg09825488 | 1 | 40974006 | EXO5 | 3.95 | 9.7x10^-5^ |  | 0.68 | 0.50 |  | 4.84 | 2.9x10^-6^ |
| cg21836919 | 1 | 207351463 |  | -2.85 | 4.6x10^-3^ |  | -4.68 | 8.6x10^-6^ |  | 1.00 | 0.32 |
| cg25631371 | 1 | 229846417 |  | -2.80 | 5.5x10^-3^ |  | -0.58 | 0.56 |  | -4.80 | 3.3x10^-6^ |
| cg21055045 | 2 | 47266465 | TTC7A | 4.20 | 3.5x10^-5^ |  | 5.02 | 2.1x10^-6^ |  | 0.87 | 0.38 |
| cg01447263 | 2 | 54366321 | ACYP2 | 2.96 | 3.3x10^-3^ |  | 4.78 | 5.8x10^-6^ |  | 0.22 | 0.82 |
| cg15739581 | 2 | 166626783 | GALNT3 | -3.34 | 9.4x10^-4^ |  | -4.79 | 5.5x10^-6^ |  | 0.52 | 0.60 |
| cg05303734 | 2 | 178101031 | NFE2L2 | -2.61 | 9.4x10^-3^ |  | -0.16 | 0.88 |  | -4.77 | 3.9x10^-6^ |
| cg00361562 | 2 | 198649771 | BOLL | -1.99 | 0.05 |  | 0.85 | 0.40 |  | -4.66 | 6.2x10^-6^ |
| cg19577697 | 2 | 232123245 | ARMC9 | 2.33 | 0.02 |  | -0.72 | 0.47 |  | 4.56 | 9.5x10^-6^ |
| cg10420726 | 4 | 71599662 | RUFY3 | 1.93 | 0.05 |  | 4.70 | 8.0x10^-6^ |  | -0.73 | 0.47 |
| cg05845376 | 5 | 140683632 | SLC25A2 | -2.77 | 5.9x10^-3^ |  | -5.04 | 2.0x10^-6^ |  | 1.05 | 0.29 |
| cg15595495 | 6 | 29798726 | HLA-G | -1.36 | 0.17 |  | -4.74 | 6.6x10^-6^ |  | 0.89 | 0.38 |
| cg00198525 | 6 | 74165863 |  | -2.30 | 0.02 |  | -4.90 | 3.5x10^-6^ |  | 0.72 | 0.47 |
| cg06002867 | 6 | 170449679 |  | -1.86 | 0.06 |  | 0.92 | 0.36 |  | -5.11 | 8.3x10^-7^ |
| cg09236008 | 7 | 23246922 |  | -2.36 | 0.02 |  | -4.84 | 4.4x10^-6^ |  | -0.29 | 0.77 |
| cg17865549 | 8 | 23541133 | NKX3-1 | -2.41 | 0.02 |  | -4.64 | 1.0x10^-5^ |  | 0.40 | 0.69 |
| cg22524508 | 11 | 13461822 | BTBD10 | -1.29 | 0.20 |  | 0.95 | 0.34 |  | -4.60 | 8.0x10^-6^ |
| cg10370850 | 11 | 70027093 | ANO1 | 3.33 | 9.7x10^-4^ |  | 4.88 | 3.8x10^-6^ |  | 1.23 | 0.22 |
| cg18714484 | 11 | 125511817 | CHEK1 | -4.09 | 5.5x10^-5^ |  | -5.01 | 2.2x10^-6^ |  | -0.38 | 0.71 |
| cg26362686 | 12 | 103125021 |  | -2.39 | 0.02 |  | -0.21 | 0.83 |  | -5.38 | 2.3x10^-7^ |
| cg25190513 | 12 | 123201362 | GPR109B | -3.09 | 2.2x10^-3^ |  | -4.68 | 8.6x10^-6^ |  | -0.22 | 0.82 |
| cg24154450 | 14 | 28851873 |  | 2.37 | 0.02 |  | 5.29 | 6.8x10^-7^ |  | -0.42 | 0.68 |
| cg05890727 | 14 | 55764647 | FBXO34 | -2.76 | 6.1x10^-3^ |  | -4.81 | 5.0x10^-6^ |  | 0.41 | 0.68 |
| cg14297991 | 14 | 97014011 | PAPOLA | 1.39 | 0.17 |  | 5.18 | 1.1x10^-6^ |  | -2.02 | 0.05 |
| cg07372700 | 15 | 31281399 | MTMR10 | 2.99 | 3.0x10^-3^ |  | 4.78 | 5.7x10^-6^ |  | -1.04 | 0.30 |
| cg21058182 | 15 | 90401931 | AP3S2 | -1.99 | 0.05 |  | 0.74 | 0.46 |  | -4.56 | 9.5x10^-6^ |
| cg14381313 | 16 | 88268339 |  | -3.68 | 2.8x10^-4^ |  | -0.12 | 0.91 |  | -5.65 | 6.4x10^-8^ |
| cg08951186 | 16 | 88290370 |  | -2.82 | 5.2x10^-3^ |  | -0.47 | 0.64 |  | -4.56 | 9.4x10^-6^ |
| cg13785068 | 17 | 3416273 |  | -2.90 | 4.0x10^-3^ |  | -0.32 | 0.75 |  | -4.57 | 9.3x10^-6^ |
| cg18493677 | 17 | 3416525 | TRPV3 | -2.63 | 8.9x10^-3^ |  | 0.04 | 0.97 |  | -4.88 | 2.3x10^-6^ |
| cg23018236 | 17 | 30244563 |  | -2.73 | 6.8x10^-3^ |  | -5.20 | 9.8x10^-7^ |  | 0.70 | 0.49 |
| cg12989128 | 20 | 42875933 | GDAP1L1 | 2.39 | 0.02 |  | -0.52 | 0.60 |  | 4.75 | 4.2x10^-6^ |
| cg22522263 | 21 | 23391891 | LOC101927843 | 1.19 | 0.23 |  | 4.92 | 3.3x10^-6^ |  | -1.97 | 0.05 |
| cg01196038 | 21 | 37985032 |  | -2.98 | 3.2x10^-3^ |  | 0.53 | 0.60 |  | -4.67 | 5.8x10^-6^ |
| cg03172077 | 22 | 22402314 |  | -2.87 | 4.4x10^-3^ |  | -4.91 | 3.4x10^-6^ |  | 1.05 | 0.29 |
| cg23304135 | X | 3187835 |  | 1.65 | 0.10 |  | 4.67 | 8.9x10^-6^ |  | -1.04 | 0.30 |

Differential methylation was carried out in the total sample, *APOE* ε4 carriers, and non-carriers

T: T-value, P: P-value

**Supplementary Table 4.** Association of AD differentially methylated CpG Sites (P<10^-5^) from *APOE* ε4 carriers and non-carriers in blood with brain imaging and cognitive traits

| **CpG Name** | **Chr** | **Position** | **Gene** | **Most Significant Global Cognitive** | |  | **Most Significant Domain-Specific Cognitive** | |  | **Most Significant Imaging** | |
| --- | --- | --- | --- | --- | --- | --- | --- | --- | --- | --- | --- |
|  |  |  |  | **Test** | **P** |  | **Test** | **P** |  | **Test** | **P** |
| cg09825488 | 1 | 40974006 | EXO5 | CDRSB | 3.3x10^-6^ |  | RAVLT_immediate | 1.9x10^-4^ |  | Hippocampal Volume | 5.6x10^-3^ |
| cg21836919 | 1 | 207351463 |  | ADAS13 | 0.01 |  | RAVLT_perc_forgetting | 2.8x10^-4^ |  | Entorhinal Thickness | 0.57 |
| cg21055045 | 2 | 47266465 | TTC7A | ADAS13 | 9.7x10^-4^ |  | RAVLT_perc_forgetting | 3.4x10^-4^ |  | Hippocampal Volume | 4.2x10^-3^ |
| cg15739581 | 2 | 166626783 | GALNT3 | ADAS13 | 1.3x10^-3^ |  | RAVLT_perc_forgetting | 4.1x10^-5^ |  | Entorhinal Thickness | 0.03 |
| cg00198525 | 6 | 74165863 |  | ADAS13 | 9.0x10^-3^ |  | RAVLT_perc_forgetting | 1.3x10^-3^ |  | Hippocampal Volume | 1.5x10^-4^ |
| cg17865549 | 8 | 23541133 | NKX3-1 | ADAS13 | 0.05 |  | RAVLT_perc_forgetting | 6.8x10^-4^ |  | Ventricle Volume | 0.06 |
| cg10370850 | 11 | 70027093 | ANO1 | ADAS13 | 3.9x10^-4^ |  | RAVLT_learning | 2.8x10^-3^ |  | Ventricle Volume | 0.06 |
| cg18714484 | 11 | 125511817 | CHEK1 | ADAS13 | 1.8x10^-5^ |  | RAVLT_perc_forgetting | 4.2x10^-5^ |  | Ventricle Volume | 8.1x10^-4^ |
| cg25190513 | 12 | 123201362 | GPR109B | ADAS13 | 7.4x10^-4^ |  | RAVLT_perc_forgetting | 1.3x10^-4^ |  | Entorhinal Thickness | 3.4x10^-4^ |
| cg05890727 | 14 | 55764647 | FBXO34 | ADAS13 | 0.08 |  | RAVLT_perc_forgetting | 3.8x10^-5^ |  | Entorhinal Thickness | 0.14 |
| cg14381313 | 16 | 88268339 |  | ADAS13 | 9.0x10^-5^ |  | RAVLT_perc_forgetting | 1.3x10^-4^ |  | Hippocampal Volume | 1.5x10^-3^ |
| cg13785068 | 17 | 3416273 |  | CDRSB | 1.4x10^-3^ |  | LDELTOTAL | 2.1x10^-3^ |  | Hippocampal Volume | 0.05 |
| cg23018236 | 17 | 30244563 |  | ADAS13 | 8.7x10^-4^ |  | RAVLT_immediate | 2.0x10^-3^ |  | Entorhinal Thickness | 0.01 |
| cg01196038 | 21 | 37985032 |  | ADAS13 | 1.1x10^-3^ |  | RAVLT_immediate | 8.0x10^-4^ |  | Ventricle Volume | 0.13 |
| cg03172077 | 22 | 22402314 |  | ADAS13 | 0.02 |  | RAVLT_perc_forgetting | 4.5x10^-4^ |  | Entorhinal Thickness | 2.3x10^-3^ |

Only CpG islands with methylation significantly (p<1.4x10^-3^) association with at least one trait shown

**Supplementary Table 5.** Association of significant differentially *APOE* methylated CpGs from the *APOE* region in FHS with cognitive traits

| **Study and CpG Name** | **Chr** | **Position** | **Gene** | **Most Significant Cognitive** | |
| --- | --- | --- | --- | --- | --- |
|  |  |  |  | **Test** | **P** |
| **Total sample:** |  |  |  |  |  |
| cg06750524 | 19 | 45409955 | APOE | trailsA | 0.16 |
| cg23270113 | 19 | 45417587 | APOC1 | trailsA | 0.01 |
| cg05644480 | 19 | 45418020 | APOC1 | PASr | 0.01 |
| **APOE e4 carriers:** |  |  |  |  |  |
| cg06750524 | 19 | 45409955 | APOE | VRd | 0.04 |
| cg23270113 | 19 | 45417587 | APOC1 | LMd | 0.05 |
| cg05644480 | 19 | 45418020 | APOC1 | SIM | 0.04 |
| **APOE e4 non-carriers:** |  |  |  |  |  |
| cg06750524 | 19 | 45409955 | APOE | LMi | 0.15 |
| cg23270113 | 19 | 45417587 | APOC1 | trailsA | 6.9x10^-3^ |
| cg05644480 | 19 | 45418020 | APOC1 | PASr | 6.7x10^-3^ |

**Supplementary Table 6. Overlapping Genes from significant co-methylated networks with Estradiol perturbation**

| **Module Name** | **Dataset** | **Genes** |
| --- | --- | --- |
| Mod2 | ROSMAP | CSF3;PLAT;KLHDC7B; CDH4;HOXA3; EPHB1; SOX5; IL15RA;TLE2;DAPK2; KRT9; CCNO; EMID2;SHC2;MFI2; LTBP3; CHST11; MBP; KCNN4; EXPH5;RAP1GAP2; PTPRN2;BTBD11; BMP6; LYPD6B; DOCK9; PIGZ; MYOM2;PITPNC1; TRIM7;NHSL1; GPR133; CAMK1D;HEG1;NAV1;MANEAL;F7; KCNMA1;GPR126;SHANK2; TNXB; PDE11A; FLNB; OPCML; C1ORF21;C15ORF27;HLA-DPB2 |
| Mod3 | ROSMAP | CSF3;PLAT;KLHDC7B;CDH4; HOXA3; EPHB1;SOX5;IL15RA;TLE2; DAPK2;KRT9;CCNO;EMID2;SHC2; MFI2;LTBP3; MBP; KCNN4;EXPH5;RAP1GAP2;PTPRN2; SLC4A11;BTBD11;BMP6;LYPD6B;CRYL1; DOCK9;PIGZ; MYOM2; TRIM7;NHSL1; GPR133; CAMK1D;HEG1; NAV1;MANEAL;F7; KCNMA1;GPR126; SHANK2;TNXB; PDE11A; FLNB;OPCML;C15ORF27; HLA-DPB2 |
| Mod4 | ROSMAP | SLC16A3;DGKZ;GRK5; STK24; VWF; NCOR2; SLC29A1; TNXB; FOXK2; PPM1F; FLNB; CYB561 |
| Mod5 | ROSMAP | NCOA4 |
| Mod8 | ADNI | PLAT;SLC16A3;DAPK2;DGKZ;GRK5;CHST11;STK24;PITPNC1;VWF;NCOA4; NCOR2;SLC29A1;SHANK2; FOXK2;PPM1F; FLNB;C1ORF21;CYB561 |

**Supplementary Figure 1.** AD differentially methylated CpGs in Blood in Brain by *APOE* genotype

**Supplementary Figure 2.** Heatmap associations with nearby gene expression for significant differentially methylated CpGs in ADNI (cg07773593)

**Supplementary Figure 3.** Methylation levels at cg07773593 in ADNI over three years

**Supplementary Figure 4.** Significant differentially (a) AD and (b) APOE methylated networks in Brain

**a**

**b**

**Supplementary Figure 5.** Significant differentially (a) AD and (b) APOE methylated networks in Blood

**a**

**b**
